# Immune Checkpoint Inhibitor-Related Myositis Overlapping With Myocarditis: An Institutional Case Series and a Systematic Review of Literature

**DOI:** 10.3389/fphar.2022.884776

**Published:** 2022-05-12

**Authors:** Yuki Nakagomi, Kazuko Tajiri, Saori Shimada, Siqi Li, Keiko Inoue, Yoshiko Murakata, Momoko Murata, Shunsuke Sakai, Kimi Sato, Masaki Ieda

**Affiliations:** ^1^ Department of Cardiology, Faculty of Medicine, University of Tsukuba, Tsukuba, Japan; ^2^ Department of Cardiology, National Cancer Center Hospital East, Kashiwa, Japan; ^3^ Department of Pharmacy, University of Tsukuba Hospital, Tsukuba, Japan; ^4^ Clinical Laboratory, University of Tsukuba Hospital, Tsukuba, Japan

**Keywords:** irAE, immune-related adverse event, cardio-oncology, onco-cardiology, troponin, creatine kinase

## Abstract

**Background:** Immune checkpoint inhibitor (ICI)-related myositis with myocarditis is a rare but potentially fatal immune-related adverse event. However, its clinical features, response to immunosuppressive treatment, and prognosis remain poorly understood. Here, we describe the clinical course of patients with ICI-related myositis overlapping with myocarditis treated at our institution and a systematic review focusing on the response to immunosuppressive therapy.

**Methods:** We identified patients who developed ICI-induced myositis with myocarditis and were treated at our hospital using a retrospective chart review of electronic medical records. For the systematic review, studies reporting ICI-induced myositis with myocarditis were identified using the Cochrane Library and PubMed databases.

**Results:** Of the 625 patients treated with ICIs, four developed myositis with concurrent myocarditis. All the patients received immunosuppressive therapy. We assessed the activity of myocarditis and myositis based on temporal changes in troponin and creatine kinase (CK) levels. In all patients, peak troponin values appeared later than the peak CK values (median, 17 days). The median time from the start of ICI therapy to the peak of troponin and CK levels was 42.5 and 28 days, respectively. In all patients, CK levels decreased rapidly and steadily after the initiation of immunosuppressants. However, troponin levels were unstable and increased. In all patients, CK levels normalized within one month (range, 12–27 days), but troponin levels took several months to normalize (range, 84–161 days). Fourteen cases of ICI-related myositis with myocarditis were included in the systematic review. Of the 14 cases, 12 (86%) had their CK level decreased after the initial steroid treatment, but the troponin level increased and was higher than that before the start of treatment. In addition, the peak troponin values appeared later than the peak CK values (a median of 6.5 days). Eight (89%) of 9 long-term follow-up patients had troponin levels above the normal range even after CK normalization.

**Conclusion:** In most cases of ICI-related myositis with myocarditis, troponin levels increased after the initial steroid treatment despite decreased CK levels, and exceeded pre-steroid levels. In addition, troponin remained elevated for several months after CK normalized.

## Introduction

Immune checkpoint inhibitors (ICIs) are a novel class of immunotherapy drugs that have improved the treatment of a broad spectrum of cancers. These drugs are being increasingly used for a large number of solid and hematological malignancies, also at early stages, and several clinical trials are underway to expand their indications (Zaha et al., 2020). To evade elimination by the host immune system, tumor cells commonly overexpress the ligands of immune checkpoint receptors, bringing T cells to a state of non-responsiveness or exhaustion (Dong et al., 2002; Wherry and Kurachi, 2015). The main effect of ICIs is to enhance a T-cell reaction against tumors. ICIs work by blocking the inhibitory pathways of T-cell activation, facilitating an effective T cell-mediated antitumor immune response. In this way, ICIs elicit an immune response primarily directed against cancer cells. Currently used ICIs include antibodies against cytotoxic T-lymphocyte-associated antigen 4 (CTLA-4), programmed cell death protein 1 (PD-1), and programmed cell death ligand 1 (PD-L1). In the tumor immunotherapy setting, ICI reactivates inactive or exhausted cytotoxic T cells, allowing them to recognize and target cancer cells. ICIs have demonstrated significant success in cancer treatment in recent years; however, the side effects associated with the use of ICIs, referred to as immune-related adverse events (irAEs), are becoming more common as these drugs become more widely used.

ICI-related myositis is rare, accounting for less than 1% of irAEs, but has a high fatality rate of up to 22% (Allenbach et al., 2020; Nguyễn et al., 2022). Interestingly, unlike idiopathic inflammatory myositis, ICI-related myositis has been reported to frequently co-occur with myocarditis in up to 40% of cases (Aldrich et al., 2021; Hamada et al., 2021). One study reported that patients with myocarditis were refractory to immunosuppressive treatment and had a 13-fold higher mortality rate than those without myocarditis (Hamada et al., 2021). These data suggest that myocarditis is a serious complication of ICI-associated myositis. Prior studies have focused on the acute presentations, management, and prognosis of patients with ICI-related myositis and myocarditis, and there are limited published reports on responsiveness to steroids and changes in myositis and myocarditis activity over time.

Here, we describe the clinical course of the four cases of ICI-related myositis with myocarditis treated at our institution, focusing on changes in myositis and myocarditis in response to immunosuppressive therapy. Moreover, we performed a systematic review of the literature to evaluate the treatment response of ICI-related myositis with myocarditis.

## Methods

### Cohort Identification and Institutional Case Series

A retrospective chart review was performed to identify patients with ICI-related myositis with myocarditis who were treated at the University of Tsukuba Hospital from January 2017 to August 2021. Data extracted from medical records included demographics, clinical presentation, medical treatment for irAEs, laboratory data, discontinuation or withholding of ICIs, and clinical outcomes. This case series is in accordance with CARE guidelines (Gagnier et al., 2013). This study was approved by our institutional review board (ethics approval number: R1-071) and the requirement for written informed consent was waived.

### Systematic Review

#### Search Strategy

To identify publications reporting treatment responsiveness in ICI-associated myositis with myocarditis, a literature search was performed based on the Preferred Reporting Items for Systematic Reviews and Meta-Analyses (PRISMA) guidelines (Moher et al., 2009; Zorzela et al., 2016). We conducted a systematic search of the PubMed and Cochrane Library databases between January 2011 and March 2022. The following terms (“myocarditis” OR “myositis” OR “troponin”) AND (“immune checkpoint inhibitor” OR “immune checkpoint inhibitors” OR “checkpoint inhibitors” OR “immune checkpoint inhibitors/adverse effects” OR “antineoplastic agents, immunological/adverse effects” OR “neoplasms/complications” OR “immunotherapy” OR “antibodies, monoclonal/therapeutic use” OR “programmed cell death 1 receptor” OR “programmed cell death” OR “CTLA-4 antigen” OR “B7-H1 antigen” OR “CTLA-4” OR “PDCD1 protein, human” OR “PD-1” OR “PD-L1”) were used in PubMed search. The search strategy for the Cochrane Library database was adapted from the PubMed database. Only articles published in English were included.

#### Selection Criteria

Case reports or case series were the articles considered for inclusion. In addition, letters or correspondence addressing relevant cases were included. Articles were considered to satisfy the inclusion criteria if they 1) reported on patients who received an ICI either as a single agent or in combination with other ICIs and developed concurrent ICI-induced myositis with myocarditis and 2) reported on the temporal changes in serum levels of creatine kinase (CK) and troponin, which indicate the activity of myositis and myocarditis, respectively.

#### Data Extraction

Two reviewers (YN and KT) independently screened the titles and abstracts of the potentially relevant articles. The full texts of eligible citations were retrieved and assessed for eligibility. All disagreements were resolved by consensus. We achieved a complete consensus before inclusion. KT extracted the following information from the articles: author, year of publication, subject age, subject sex, type of cancer, type of ICI, in-hospital outcome, treatment for irAEs, time to peak CK or troponin level from ICI initiation, and responsiveness to irAE treatment. This information was checked for accuracy by YN.

## Results

### Summary of Institutional Case Series

To understand the treatment responsiveness of ICI-associated myositis with myocarditis overlap syndrome, we first examined our institutional database. In total, 625 patients were treated with ICIs at our hospital between January 2017 and August 2021. Among these, 583 patients were treated with a PD-1 or PD-L1 inhibitor alone and 42 were treated with a PD-1 inhibitor and CTLA-4 inhibitor combination. There were four cases of ICI-related myositis with myocarditis (incidence 0.64%), consisting of three men and one woman with a median age of 75 years (range, 46–80 years) (Table 1). All four patients were diagnosed with myocarditis based on the definitions suggested by Bonaca et al. (Bonaca et al., 2019). Three patients (cases 1, 3, and 4) had tissue pathology suggestive of myocarditis on endocardial biopsy, and one patient (case 2) had a new wall motion abnormality on echocardiogram unexplained by other diagnoses and all of the following: clinical syndrome consistent with myocarditis, elevated troponin levels, ECG abnormality, and negative coronary angiography. The diagnosis of myositis in patients of cases 1–3 was based on elevated CK levels, myalgia, and electromyography (EMG) and magnetic resonance imaging (MRI) findings, which were consistent with myositis. Case 4 patient was clinically diagnosed with myositis owing to elevated CK levels. Two patients (cases 2 and 3) had myasthenia gravis (MG). The median number of ICI cycles administered was one (range, 1–2). The details of these cases are described in the following sections.

**TABLE 1:** Demographics and patient information of our institutional cases.

Case	Age	Gender	Malignancy	ICI	Number of ICI Cycles	irAEs (CTCAE Grade)	In-Hospital Outcome
1	46	F	Gastric cancer	Nivolumab	1	Myocarditis (G1), Myositis (G2)	Alive
2	77	M	RCC	Pembrolizumab	1	Myocarditis (G1), Myositis (G2), MG (G2)	Alive
3	73	M	RCC	Nivolumab	2	Myocarditis (G2), Myositis (G2), MG (G2)	Alive
4	80	M	Bladder cancer	Durvalumab	1	Myocarditis (G1), Myositis (G1)	Alive

CTCAE, common terminology criteria for adverse events; G, grade; ICI, immune checkpoint inhibitor; irAE, immune-related adverse events; MG, myasthenia gravis; RCC, renal cell carcinoma.

**FIGURE 1:**
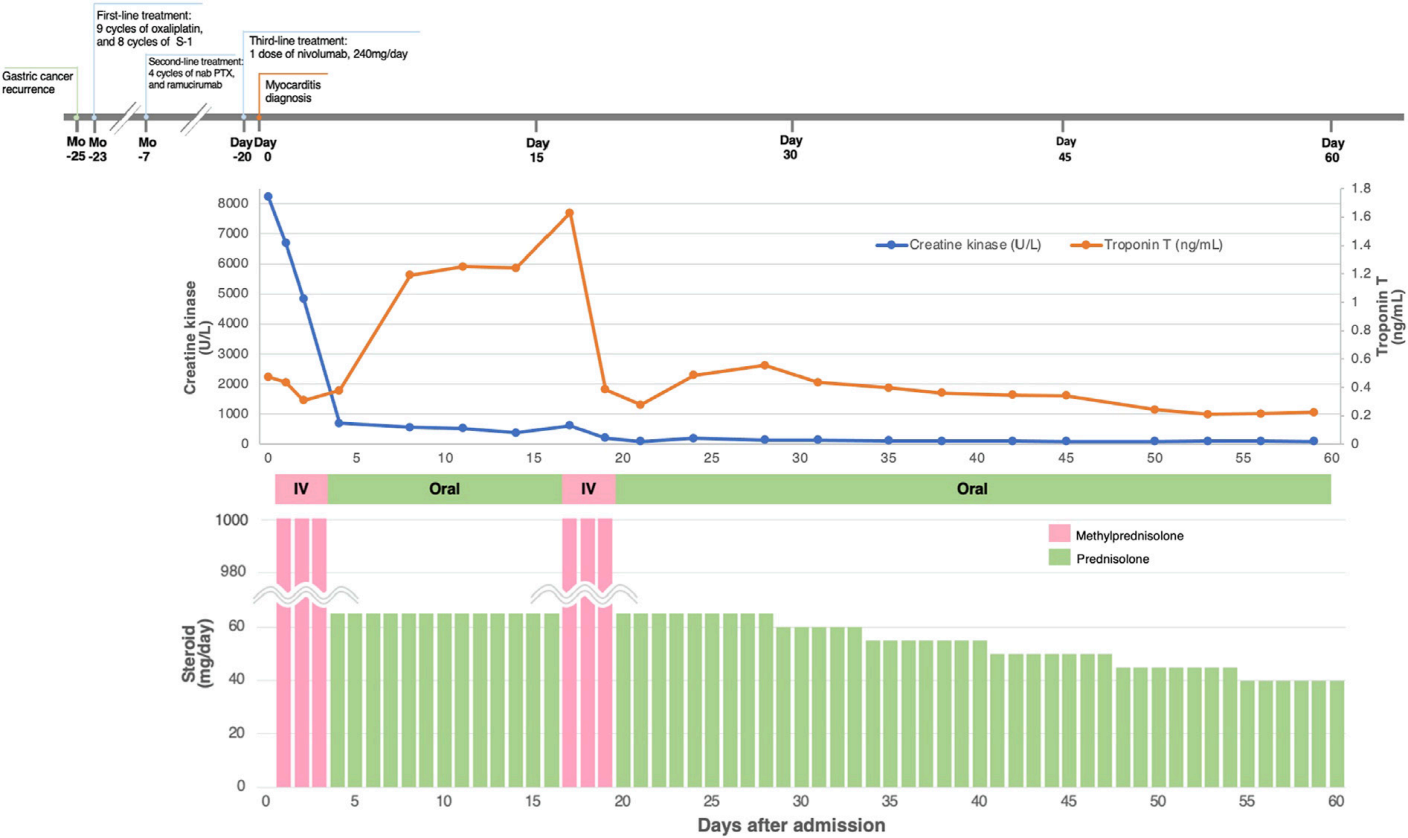
Clinical course and changes in CK and troponin in case 1.

**FIGURE 2:**
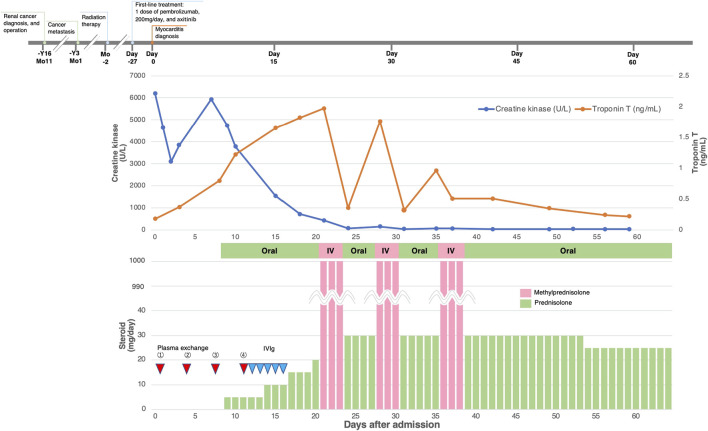
Clinical course and changes in CK and troponin in case 2.

**FIGURE 3:**
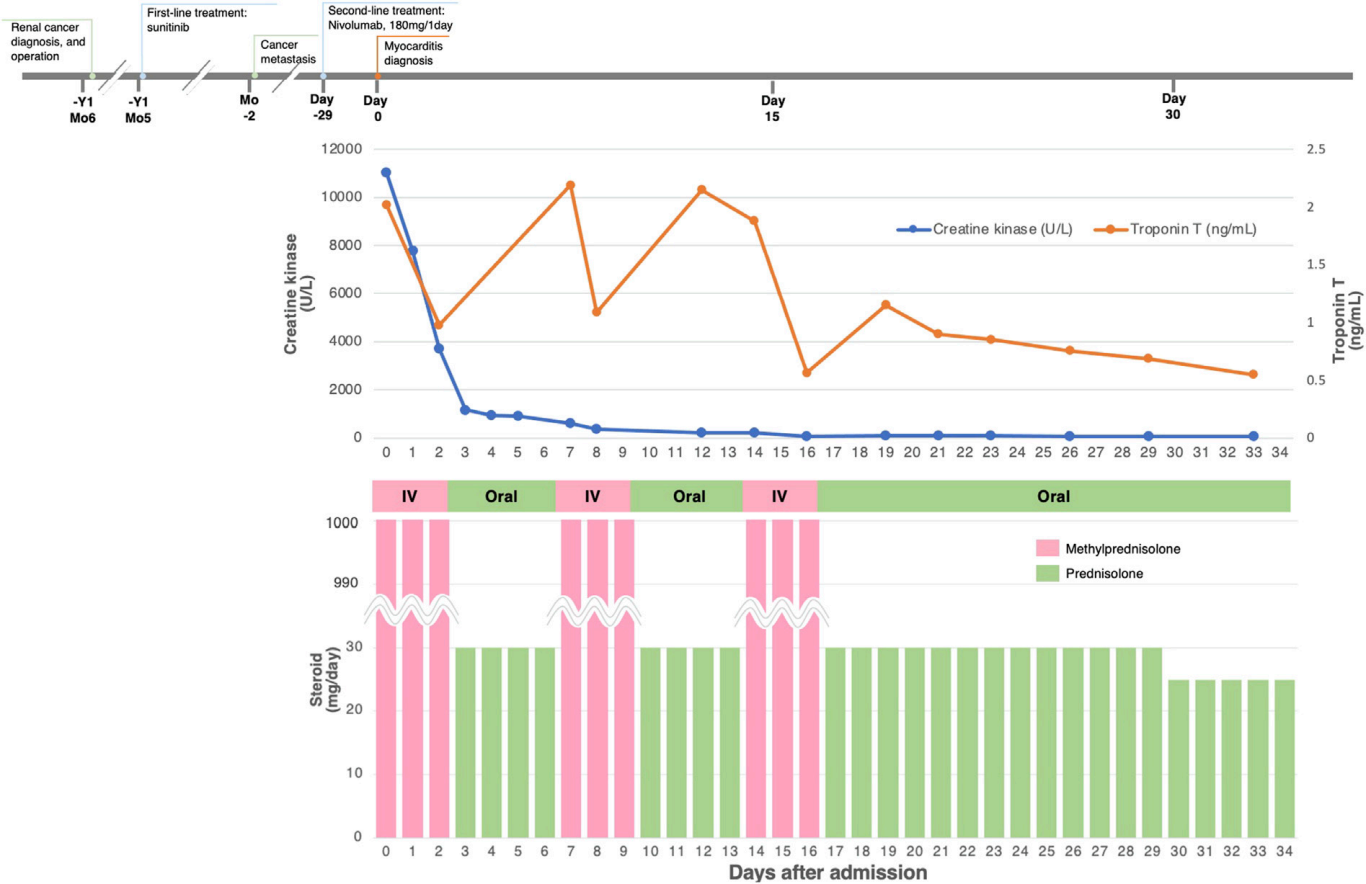
Clinical course and changes in CK and troponin in case 3.

**FIGURE 4:**
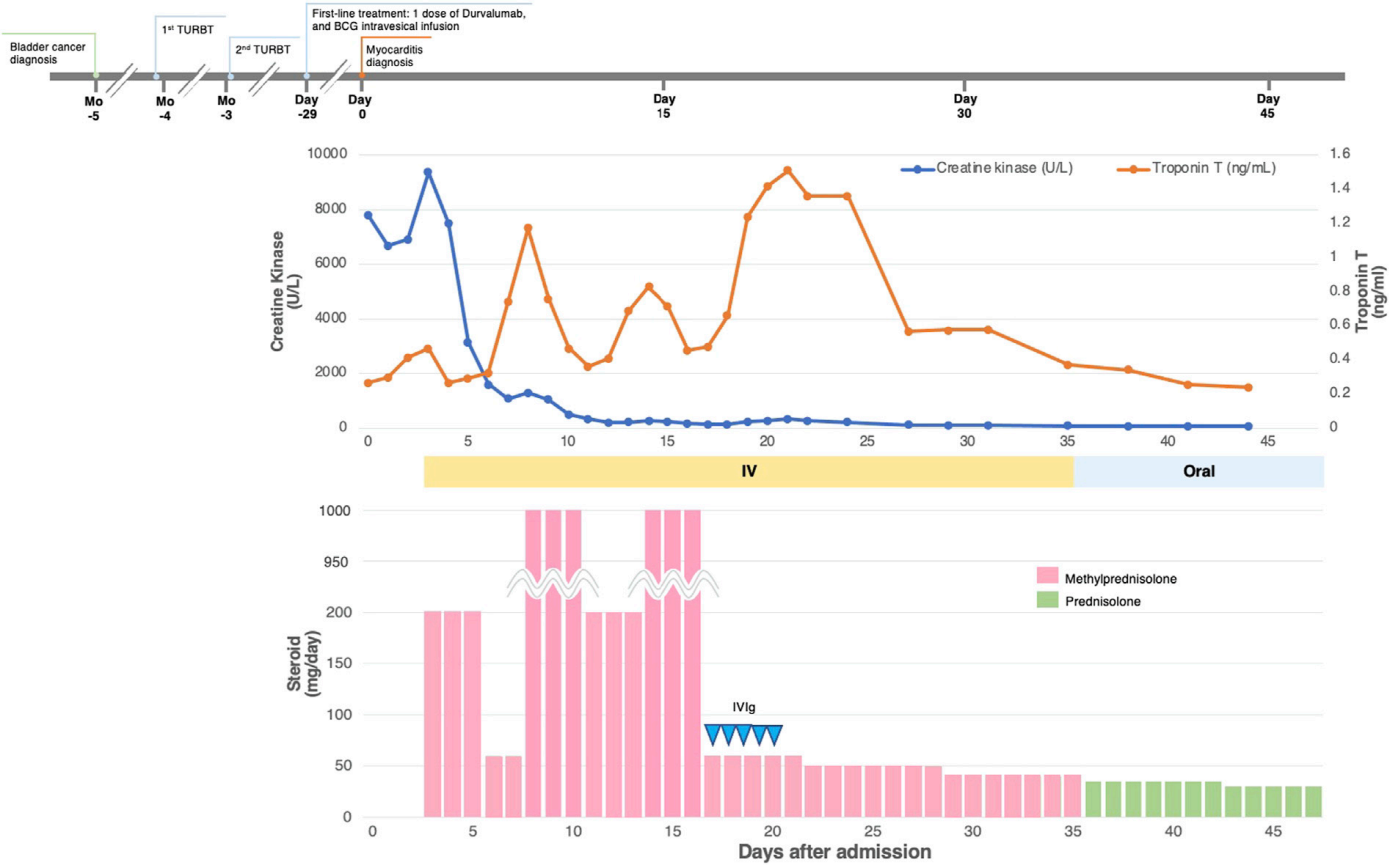
Clinical course and changes in CK and troponin in case 4.

All the patients received immunosuppressive therapy (Table 2; Figures 1–4). Two patients (cases 1 and 3) were started on high-dose steroids (methylprednisolone [mPSL] 1 g), one patient (case 4) on medium-dose steroids (mPSL 200 mg), and one patient (case 2) on plasma exchange, intravenous immunoglobulin (IVIG), and low-dose oral prednisolone (PSL), followed by steroid pulse therapy. We assessed the activity of myocarditis and myositis based on the temporal changes in troponin and CK levels. As shown in Figures 1–4, the time kinetics of troponin were clearly different from those of CK. In all patients, peak troponin values appeared later than peak CK values (median of 17 days [range, 7–21]) (Table 2; Figures 1–4).The median time from the start of ICI treatment to the peak of troponin and CK levels was 42.5 and 28 days, respectively. In all patients, the CK levels decreased rapidly and steadily after the initiation of steroid therapy. However, the troponin levels were unstable and increased before the initiation of steroids. After repeated steroid pulse therapy with or without IVIG, the troponin levels began to decrease steadily. The median time from the start of immunosuppressive therapy to a 90% reduction in CK was 7 days (range, 4–20) (Table 2). In contrast, the time to 90% reduction in troponin was considerably longer (range, 63–161 days) than that in CK (Table 2). In all patients, CK levels normalized within one month (range, 12–27 days), but troponin levels took several months (range, 84–161 days) to decrease to within the normal limits (Table 2). These results indicate that in patients with ICI-associated myositis with overlapping myocarditis, myocarditis was more resistant to immunosuppressive therapy than myositis.

**TABLE 2:** Treatment responsiveness of our institutional cases.

Case	Daily dose of Corticosteroid	Other ISTs	Time to peak CK from ICI Start (days)	Time to peak Troponin from ICI Start (days)	Time to 90% reduction in CK from IST Start (days)	Time to 90% reduction in Troponin from IST Start (days)	Time to normalize in CK from IST Start (days)	Time to normalize in Troponin from IST Start (days)
1	mPSL 1 g	None	21	37	7	63	27	84
2	mPSL 1 g	PE, IVIG	27	48	20	>62^a^	23	>62^a^
3	mPSL 1 g	None	29	36	4	69	12	139
4	mPSL 200 mg then increased to 1 g	IVIG	32	50	7	161	21	161

a Follow-up of this patient was terminated before the troponin level dropped below 90% of the peak value or was normalized.

CK, creatinine kinase; ICI, immune checkpoint inhibitor; IST, immunosuppressive therapy; IVIG, intravenous immunoglobulin; mPSL, methylprednisolone; PE, plasma exchange.

#### Case 1

The patient was a 46-year-old woman with metastatic gastric cancer who was treated with nivolumab as third-line therapy. She had undergone thymectomy for MG 23 years prior. Fourteen days after the first dose, a second dose of nivolumab was withheld due to elevated liver transaminase levels. Twenty days after her first dose, she complained of neck pain and bilateral shoulder and lower extremity pain with elevated levels of serum CK (8,236 U/L [normal range, 42–150 U/L]) and troponin T (0.474 ng/ml [normal level, <0.1 ng/ml]) (Figure 1). The patient was admitted to our hospital with suspected myocarditis and myositis. She did not report any chest symptoms. Her electrocardiogram (ECG) showed a T-wave abnormality, which has not been previously documented. Echocardiography revealed a normal left ventricular ejection fraction (LVEF), and brain natriuretic peptide (BNP) level was within the normal limits [normal level, <18.4 pg/ml]). An endomyocardial biopsy was performed one day after admission. Histopathological analysis of the biopsy samples revealed strong infiltration of CD4^+^ T cells, CD8^+^ T cells, and CD68^+^ histiocytes, with mild infiltration of CD20^+^ B lymphocytes (Supplementary Figure S1). Her EMG findings were consistent with myositis. She was diagnosed with myocarditis and myositis and nivolumab was discontinued. She was treated with 3 days of steroid pulse therapy with intravenous mPSL at a dose of 1 g/day, followed by oral PSL at 1 mg/kg/day. Immediately after steroid pulse therapy, she recovered from myositis as CK decreased and myalgia disappeared, but myocarditis activity increased as indicated by an increase in troponin level (Figure 1). Therefore, she received steroid pulse therapy for more than 3 days with 1 g/day of mPSL. After therapy, her troponin level declined, and the steroid dose was tapered step-by-step. During hospitalization, her LVEF remained normal and no arrhythmia occurred. The patient was discharged after 60 days of hospitalization. She was treated with three cycles of trifluridine/tipiracil with ramucirumab, which resulted in further disease progression. Four months after discharge, the patient opted to transition to hospice care.

#### Case 2

A 77-year-old man with metastatic renal cell carcinoma (RCC) was treated with pembrolizumab. The patient had a history of unstable angina. Twenty-one days after his first dose, he developed hoarseness and back pain with an elevated CK level (1,177 U/L [normal range, 63–257 U/L]), and the second dose of pembrolizumab was withheld. Twenty-seven days after the first dose, worsening hoarseness, ptosis, diplopia, diffuse body pain, and lightheadedness appeared, and he was admitted to our hospital. He did not complain of chest symptoms. Blood tests showed elevated troponin T (0.176 ng/ml) and CK (6,197 U/L) levels. ECG revealed a newly developed T-wave abnormality. Echocardiography revealed a slight decrease in LVEF to 55% and wall motion abnormality in the apex. BNP level was slightly elevated (55.8 pg/ml). The result of the edrophonium test was positive. His MRI findings were consistent with myositis. The patient was diagnosed with myocarditis concomitant with myositis and MG. He was started on plasma exchange, followed by IVIG (400 mg/kg/day for 5 days) with uptilting of the oral PSL (Figure 2). After the initiation of immunosuppressive therapy, symptoms of MG and myositis improved and CK levels declined in response to the therapy, but the activity of myocarditis worsened (Figure 2). Therefore, the patient received steroid pulse therapy with intravenous mPSL at 1 g/day for 3 days. Immediately after the therapy, the troponin level decreased, but soon thereafter, it re-elevated. Therefore, a second steroid pulse therapy was performed. However, the troponin level then increased again. A third steroid pulse therapy was administered and the troponin level decreased stably. During hospitalization, the LVEF did not worsen and no arrhythmia occurred. He was transferred to another hospital for rehabilitation on the 59th day of his hospital stay.

#### Case 3

The patient was a 73-year-old man with metastatic RCC who received two cycles of nivolumab as second-line therapy. The patient had a history of anterior wall myocardial infarction. On the 15th day after the first dose, he developed chest pain, bilateral thigh muscle pain, and ptosis of the right eyelid. Twenty-nine days after the first administration of nivolumab, he was admitted to our hospital because of worsening symptoms. He had elevated levels of serum CK (11,021 U/L [normal range, 63–257 U/L]) and troponin T (2.02 ng/ml) (Figure 3). His ECG showed multiple premature ventricular contractions with advanced atrioventricular block, which has not been previously documented. Echocardiography showed that the LVEF was mildly diminished (45%) with an akinetic apex wall, which was also observed before ICI initiation. His BNP level was elevated (332.2 pg/ml). Coronary angiography was done to exclude relevant coronary diseases. Histopathological analysis of endomyocardial biopsy specimens revealed an intense and patchy infiltrate of CD68^+^ macrophages and CD3^+^ lymphocytes (especially CD8^+^ T cells) in the myocardium (as previously reported (Sakai et al., 2020)). His MRI findings were consistent with myositis. The patient was diagnosed with myocarditis concomitant with myositis and MG, and nivolumab was discontinued. He received pulse therapy with intravenous mPSL of 1 g/day for 3 days, followed by oral PSL. After steroid pulse therapy, the patient quickly recovered from myositis and MG as CK decreased and his eyelid ptosis improved, but myocarditis activity increased, as indicated by elevated troponin levels and persistent chest discomfort (Figure 3). Therefore, two more rounds of steroid pulse therapy were needed to reduce the inflammatory activity in the myocardium. The patient was discharged on day 36 of hospitalization. He was treated with axitinib (third-line therapy), followed by everolimus (fourth-line therapy), which resulted in further cancer progression. Seven months after myocarditis development, the patient died of hemorrhagic cerebral infarctions due to a left ventricular mobile thrombus (as previously reported (Sakai et al., 2020)).

#### Case 4

The patient was an 80-year-old man with bladder cancer who was treated with durvalumab. He had a history of myocardial infarction, diabetes mellitus, and dyslipidemia. Twenty-five days after his first dose, his liver transaminase levels were elevated; therefore, a second dose of durvalumab was withheld. Twenty-nine days after the first dose, troponin T (0.268 ng/ml) and CK (7,815 U/L [normal range, 63–257 U/L]) were also found to be elevated. Although he did not complain of chest pain or other symptoms, he was admitted to our hospital with suspected myocarditis and myositis. ECG revealed a newly developed T-wave abnormality. Echocardiography revealed a mild decrease in LVEF (45%) with local wall motion abnormalities, but this was unchanged from the echocardiography done prior to ICI administration. His BNP level was slightly elevated (91.2 pg/ml). Endomyocardial biopsy revealed moderate to severe inflammatory cell infiltration, consisting mainly of lymphocytes, and cardiomyocyte shedding. Lymphocytes were a mixture of CD3^+^ T cells and CD20^+^ B cells with infiltration of CD68^+^ macrophages (Supplementary Figure S2). He was diagnosed with myocarditis and myositis and durvalumab was discontinued. The patient was initially treated with steroid pulse therapy with 200 mg of intravenous mPSL for 3 days, followed by 1 mg/kg/day of mPSL. However, as shown in Figure 4, troponin levels significantly increased despite a decrease in CK levels (Figure 4). Therefore, the patient received two more rounds of pulse therapy with an increased dose of mPSL (1 g/day) and IVIG (400 mg/kg/day for 5 days). After therapy, his troponin level declined, and the steroid dose was tapered step-by-step. During hospitalization, his LVEF was stable and no arrhythmia occurred. The patient was discharged 47 days after hospitalization. The patient has been recurrence-free since then. However, two years after discharge, he was diagnosed with metastatic prostate cancer and died six months later.

### Systematic Review

**FIGURE 5:**
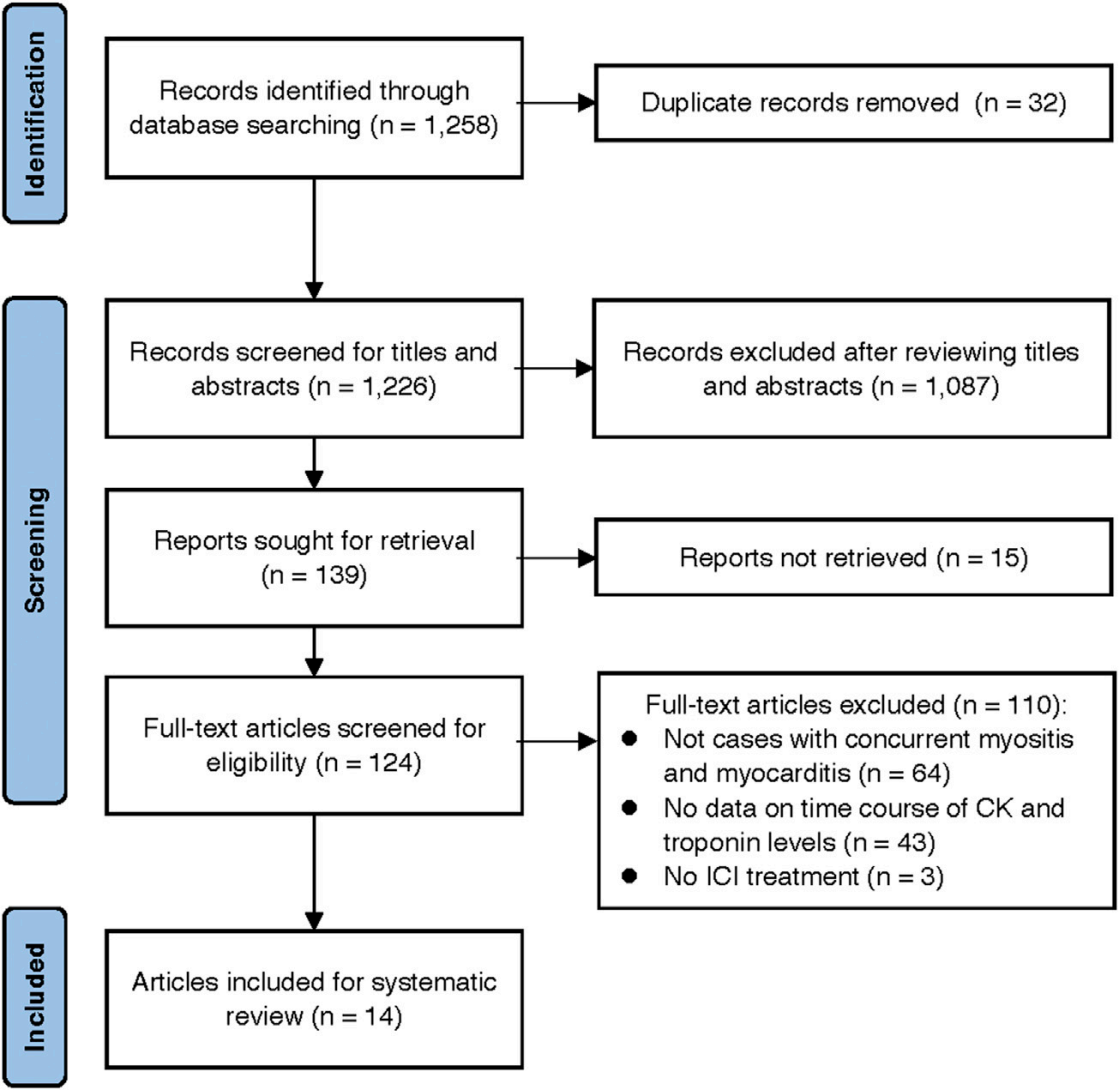
Preferred Reporting Items for Systematic Reviews and Meta-Analyses (PRISMA) flowchart of the study selection process.

After a review of 1,258 citations in PubMed and the Cochrane Library, 14 case reports, including 14 patients, met the selection criteria of our systematic review (Saibil et al., 2019; Salem et al., 2019; Shirai et al., 2019; Giancaterino et al., 2020; Lie et al., 2020; Matsui et al., 2020; Todo et al., 2020; Xing et al., 2020; Cao et al., 2021; Deng et al., 2021; Hu et al., 2021; Jespersen et al., 2021; Yang et al., 2021; Shindo et al., 2022) (Figure 5). The clinical characteristics and prognosis of the 14 patients are summarized in Table 3. The median age was 68 years (range, 33–88 years), and the majority of patients were men (13/14 [93%]). The median number of ICI cycles was one (range, 1–3). Three patients (21%) died from irAEs.

**TABLE 3:** Clinical characteristics and prognosis of previously reported cases.

Author, year, Ref. #	Age	Gender	Malignancy	ICI	Number of ICI Cycles	CV Toxicity	Myositis	Other irAEs	In-Hospital Cause of death
Cao et al. (2021)	69	M	Esophagogastric junction carcinoma	Pembrolizumab	1	Myocarditis, CHB	+	SJS/TEN, liver	(Alive)
Deng et al. (2021)	71	M	Lung cancer	Tislelizumab	1	Myocarditis	+	Lung, liver, pituitary	(Alive)
Giancaterino et al. (2020)	88	M	Melanoma	Nivolumab	1	Myocarditis, CHB, VF, cardiac arrest	+	None	Cardiac death
Hu et al. (2021)	66	M	UTUC	Tislelizumab	1	Myocarditis, CHB	+	Liver, kidney	(Alive)
Jespersen et al. (2021)	57	M	Renal cell carcinoma	Nivolumab + ipilimumab	1	Myocarditis, CHB, VT, cardiac arrest	+	MG	(Alive)
Lie et al. (2020)	79	M	Pleural mesothelioma	Nivolumab	2	Myocarditis	+	None	(Alive)
Matsui et al. (2020)	69	M	UTUC	Pembrolizumab	2	Myocarditis, DHF, cardiomyopathy, CHB, cardiac arrest	+	None	Cardiac death
Saibil et al. (2019)	67	M	Melanoma	Nivolumab + ipilimumab	1	Myocarditis, CHB, cardiomyopathy	+	None	Cardio-respiratory failure
Salem et al. (2019)	66	F	Lung cancer	Nivolumab	3	Myocarditis	+	None	(Alive)
Shindo et al. (2022)	79	M	Gastric cancer	Nivolumab	1	Myocarditis	+	None	(Alive)
Shirai et al. (2019)	83	M	Melanoma	Pembrolizumab	1	Myocarditis	+	MG	(Alive)
Todo et al. (2020)	63	M	Bladder carcinoma	Pembrolizumab	2	Myocarditis	+	MG	(Alive)
Xing et al. (2020)	66	M	Lung cancer	Sintilimab	2	Myocarditis, CHB	+	MG	(Alive)
Yang et al. (2021)	33	M	Thymoma	Sintilimab	1	Myocarditis, cardiomyopathy	+	MG	(Alive)

CHB, complete heart block; CV, cardiovascular; DHF, decompensated heart failure; ICI, immune checkpoint inhibitor; MG, myasthenia gravis; SJS/TEN, Stevens-Johnson syndrome/toxic epidermal necrolysis; UTUC, upper tract urothelial carcinoma; VF, ventricular fibrillation; VT, ventricular tachycardia.

All 14 patients received immunosuppressive therapy starting with steroids (Table 4). After the initial steroid treatment, CK levels decreased in all patients. However, in 12 patients (86%), troponin levels increased after the initial steroid treatment and exceeded the level before the start of therapy. In addition, their peak troponin values appeared later than the peak CK values (median, 6.5 days [range, 1–23]). In seven of the 14 patients (50%), the steroid doses had to be increased since no clinical improvement was observed. Eleven patients (79%) also received immunosuppressive therapy other than steroids such as mycophenolate mofetil, infliximab, abatacept, IVIG, and plasma exchange. In nine of the 14 cases, CK values were assessed over time until they fell within the normal range. Eight of the 9 patients (89%) had troponin levels above the normal range, even after CK normalization.

**TABLE 4:** Treatment responsiveness of previously reported cases.

Author, year, Ref. #	Daily dose of Corticosteroid	Other ISTs	Troponin T or I	Time to peak CK from ICI Start (days)^a^	Time to peak Troponin from ICI Start (days)^a^	Time Course of CK and Troponin values after IST initiation^a^
Cao et al. (2021)	mPSL 0.5 g	IVG, PE	T	On admission (after 1 cycle of ICI)	1 day after CK peaked	CK declined to WNL by 1 week; troponin gradually decreased but remained elevated at 40 days
Deng et al. (2021)	Prednisone 20 mg increased to mPSL 80 mg	None	T	36	46	CK declined sharply and steadily; troponin was re-escalated during tapering of steroids
Giancaterino et al. (2020)	Prednisone 40 mg increased to mPSL 1 g	Infliximab	T	23	26	CK steadily declined in response to initial non-high-dose steroids; however, troponin was elevated and remained at a high level
Hu et al. (2021)	mPSL 0.5 mg/kg increased to 1.5 mg/kg	IVIG	I	21	27	CK declined in response to initial mild-dose steroids; however, troponin increased further, requiring intensive IST.
Jespersen et al. (2021)	mPSL 2 mg/kg increased to 1 g	Abatacept, MMF	I	12	18	CK declined in response to initial non-high-dose steroids; however, troponin increased further, requiring intensive IST. CK and troponin normalized almost simultaneously after 1 month
Lie et al. (2020)	mPSL 1 g	MMF	T	On admission (after 2 cycles of ICI)	13 days after CK peaked	CK declined sharply to WNL by day 20; troponin was re-escalated during tapering of steroids and remained elevated at 1 month
Matsui et al. (2020)	mPSL 15 mg increased to 1 g	PE	I	26	30	CK decreased to WNL within 1 week in response to initial mild steroids and PE; however, troponin increased further, requiring SPT. Troponin remained elevated until the day of cardiac death, 15 days after IST initiation
Saibil et al. (2019)	mPSL 200 mg increased to 1 g	Infliximab, IVIG	I	16	16	CK declined sharply and steadily after SPT; however, troponin was re-escalated and remained elevated until the day of death, 2 weeks after IST.
Salem et al. (2019)	mPSL 0.5 g	PE, abatacept	T	48	56	CK declined rapidly in response to SPT; however, troponin increased, requiring PE and abatacept. CK normalized after 1 month, but troponin remained elevated at 3.5 months
Shindo et al. (2022)	Prednisolone 30 mg	None	T	16	35	CK declined rapidly in response to steroids; however, troponin increased. CK normalized after 3 weeks, but troponin remained elevated at 13 weeks
Shirai et al. (2019)	mPSL 1 g	PE	T	29	36	CK declined rapidly in response to IST; however, troponin increased and remained elevated at 1 month
Todo et al. (2020)	Prednisolone 60 mg	None	I	40	40	CK responded to IST and normalized within 1 month; troponin remained elevated at 2 months
Xing et al. (2020)	mPSL 2 mg/kg increased to 0.5 g	IVIG, PE	T	31	54	CK responded to IST and normalized within 1 month; troponin was re-escalated during tapering of steroids and remained elevated at 11 weeks
Yang et al. (2021)	mPSL 2 mg/kg	IVIG	T	30	40	CK responded to IST and normalized within 5 months; Troponin was re-elevated during steroid administration and remained high at 5 months

a This was inferred from the text and graphs displayed in the manuscript.

CK, creatinine kinase; ICI, immune checkpoint inhibitor; IST, immunosuppressive therapy; IVIG, intravenous immunoglobulin; MMF, mycophenolate mofetil; mPSL, methylprednisolone; PE, plasma exchange; SPT, steroid pulse therapy; WNL, within normal limits.

## Discussion

In this institutional case series and systematic review of ICI-related myositis overlapping with myocarditis, there were two major findings. First, in most cases, troponin levels peaked later than the CK levels. The CK levels decreased rapidly and steadily after the initiation of steroid therapy. However, the troponin levels were unstable and even increased after the initiation of steroids. Second, in the majority of cases, troponin continued to rise on immunosuppressive treatment even while CK showed a decreasing trend and remained elevated for several months after CK normalized. These results indicate that myocarditis is more resistant to immunosuppressive therapy in patients with ICI-associated myositis overlapping with myocarditis.

An important finding of this study was the difference in treatment response between myositis and myocarditis, as indicated by the different behaviors of CK and troponin over time. There was no correlation between CK and troponin levels, which peaked at different times. In many cases, troponin levels remained elevated even after CK normalized. This discordance could be due to the fact that myocarditis is more resistant to immunosuppressive treatment than myositis. Considering that myocarditis may be refractory to treatment, a careful long-term follow-up with troponin is required. Few cases have reported changes in troponin levels over time in patients with ICI-related myocarditis alone (Norwood et al., 2017; Ida et al., 2022; Osinga et al., 2022). In these cases, high troponin levels were found to prolong after the initiation of steroid therapy. These findings suggest that ICI myocarditis with or without concomitant myositis might be refractory to immunosuppressive treatment and prolonged troponin elevation. In addition, longer and larger prospective cohort studies are needed to answer this issue.

For severe cases with grade 3–4 ICI-related myositis, cancer society guidelines recommend starting with prednisone 0.5–1 mg/kg or equivalent, and consideration of intravenous mPSL 1–2 mg/kg or higher-dose bolus if severely compromised (Haanen et al., 2017; Brahmer et al., 2018). In contrast, for ICI-related myocarditis, many cardiologists use higher doses as initial therapy, as recommended by several panels (Curigliano et al., 2020; Palaskas et al., 2020; Thompson et al., 2020; Lehmann et al., 2021; Thuny et al., 2021), similar to the doses used in transplant allograft rejection (i.e., intravenous mPSL 1 g for 3 days). A retrospective study of 126 patients with ICI-related myocarditis revealed an inverse relationship between the initial corticosteroid dose and the occurrence of major adverse cardiac events (MACE) (Zhang et al., 2020). Compared with low-dose corticosteroids (<60 mg/day of mPSL or equivalent), a high dose (0.5–1 g/day) was associated with a 73% lower risk of MACE (Zhang et al., 2020). Moreover, patients who received steroid therapy within 24 h were less likely to have persistent troponin elevation at discharge than those who received treatment after 24 h (Zhang et al., 2020). Thus, timely intervention with high-dose corticosteroid therapy appears to be important for reducing myocardial damage and improving cardiac outcomes. In this study, 12 patients (two from our institutional case series and 10 from the systematic review) were started on non-high-dose steroids, but in nine of them (two from our case series and seven from the systematic review), the dose of steroids was eventually increased due to disease progression. Since high-dose steroids are often required in cases of ICI-related myositis complicated by myocarditis, it is reasonable to preferentially begin with high-dose corticosteroids to control disease activity.

Although steroids are the first-line treatment for ICI-related myocarditis, there is a lack of evidence regarding optimal subsequent treatment for patients who are refractory to steroid therapy. Antithymocyte globulin, infliximab, mycophenolate, IVIG, abatacept, and plasmapheresis are recommended (Curigliano et al., 2020; Thompson et al., 2020), but the data is mostly based on anecdotal experience. In this study, 13 patients (two from our case series and 11 from the systematic review) received immunosuppressive agents other than steroids. Among them, only one case report showed that supplementary immunosuppressive agents (abatacept and mycophenolate) effectively suppressed the activity of both myositis and myocarditis, and both CK and troponin levels decreased almost simultaneously to within normal limits within a month (Jespersen et al., 2021). However, in most of the 13 cases, troponin levels remained elevated, even after a steady decrease in CK levels. The optimal treatment for ICI-related myositis with myocarditis is unknown, but these patients seem to require intensive, long-term immunosuppressive therapy. Owing to concerns about the side effects of long-term steroid administration, the use of immunosuppressive drugs other than steroids should be considered.

Our study had some limitations. First, this was a retrospective study in nature. The timing, interval, and duration of the CK and troponin measurements varied from case to case. Second, in some cases in the systematic review, CK and troponin values or the timing of their peaks or normalizations were inferred from figures in the articles. Further research in larger cohorts that prospectively assess changes in these markers over time is needed.

## Conclusion

In most cases of ICI-related myositis with myocarditis, troponin levels increased after the initial steroid treatment despite decreased CK levels, and exceeded pre-steroid levels. In addition, troponin remained elevated for several months after CK normalized.

## Data Availability

The raw data supporting the conclusion of this article will be made available by the authors, without undue reservation.
