# Supplementary material for: Immune Checkpoint Inhibitor-Related Myositis Overlapping With Myocarditis: An Institutional Case Series and a Systematic Review of Literature
Source: Front Pharmacol. 2022 May 12;13:884776. doi: 10.3389/fphar.2022.884776 (PMC9135130; doi:10.3389/fphar.2022.884776)
Supplement: Supplementary file 1 [file DataSheet1.docx]

Supplementary Material

## Supplementary Figures

**Supplementary Figure 1. Histological analysis of endomyocardial biopsy from case 1.** Scale bar = 50 μm.

**Supplementary Figure 2. Histological analysis of endomyocardial biopsy from case 4.** Scale bar = 50 μm.
